# Corrigendum

**DOI:** 10.1111/pbi.13423

**Published:** 2020-06-12

The authors of Sun *et al. *(2020) have identified some errors in Tables S3, S4 and S5 after original publication. These Tables have been amended in the online version of the article.
